# Decision-Making in Pediatric Hematopoietic Stem Cell Transplantation: Perspectives of Physicians, Patients, and Families

**DOI:** 10.7759/cureus.107167

**Published:** 2026-04-16

**Authors:** Luisa Arenas-Pérez, Andres F Calderon A, Diana Estrada

**Affiliations:** 1 Pediatrics, Pontificia Universidad Javeriana, Bogotá, COL; 2 Pediatrics, Pontificia Universidad Javeriana, Bogota, COL; 3 Pediatric Oncology, Instituto Nacional de Cancerología, Bogotá, COL; 4 Pediatrics, Hospital Universitario San Ignacio, Bogotá, COL

**Keywords:** decision-making, family perspectives, hematopoietic stem cell transplantation, medical ethics, pediatric patients, shared decision-making

## Abstract

Pediatric hematopoietic stem cell transplantation (HSCT) is a complex therapeutic procedure that presents significant ethical, clinical, and psychosocial challenges. Decision-making in this context involves multiple stakeholders, including physicians, patients, and families, and is influenced by several medical and non-medical factors. This narrative review provides a descriptive synthesis of the literature to analyze the factors influencing decision-making in pediatric HSCT and to explore the roles and perspectives of the different actors involved in this process. Relevant literature was identified through searches of electronic databases, including PubMed, Scopus, Embase, and Web of Science, including studies published up to March 2024. Gray literature was not systematically included. The findings highlight that clinical condition, availability and understanding of information, socioeconomic context, religious beliefs, and perception of risks and benefits significantly influence treatment decisions. In addition, the perspectives of patients, parents, and healthcare professionals play a critical role in shaping the decision-making process. Shared decision-making, clear communication, and adequate psychological support are essential elements to facilitate informed and ethically sound decisions. Understanding these dynamics is fundamental to improving patient-centered care in pediatric transplantation.

## Introduction and background

Hematopoietic stem cell transplantation (HSCT) is a medical procedure based on the collection of hematopoietic stem cells, the administration of a conditioning regimen, and the infusion of these cells into a recipient to generate a new hematopoietic and immune system, effectively replacing the recipient’s hematopoietic system with donor-derived cells [1,2]. This procedure is indicated in the management of multiple hematologic, immunologic, and autoimmune diseases, as well as certain inborn errors of metabolism.

HSCT can be classified according to the origin of the donor cells into autologous transplantation, in which the patient serves as both donor and recipient; allogeneic transplantation, in which the donor is a different individual; and syngeneic transplantation, in which the donor and recipient are identical twins. It may also be classified according to the source of hematopoietic stem cells, including bone marrow, peripheral blood, and umbilical cord blood [3].

Over the past decades, the number of HSCTs performed worldwide has increased substantially, and a significant proportion of these procedures are performed in children, adolescents, and young adults under 20 years of age. Since the first successful HSCT was performed in 1968, considerable advances in transplant medicine have led to cure rates approaching 70% in selected patients. HSCT is currently considered a potentially curative therapy for a wide range of malignant and non-malignant pediatric conditions, including leukemias, bone marrow failure syndromes, and primary immunodeficiencies. However, despite improvements in outcomes, the procedure remains complex and high-risk, requiring careful evaluation of both expected survival and treatment-related morbidity. These considerations are particularly relevant in pediatric populations, where long-term sequelae and quality of life play a critical role in treatment decisions [4].

Despite these advances, HSCT remains associated with substantial morbidity and mortality. Reported survival rates range from approximately 60% to 70%, while morbidity can reach up to 60% due to chronic health conditions such as developmental disorders, cognitive impairment, behavioral alterations, and severe transplant-related complications. Although transplantation offers the best and sometimes the only chance of cure or long-term survival for many children with malignant and non-malignant disorders, it also raises numerous ethical and medical challenges, including balancing risks and benefits for both donor and recipient, integrating emerging medical technologies, and navigating the complex social context in which treatment decisions are made [4,5].

## Review

Search strategy and study selection

A narrative review of the literature was conducted to identify studies addressing decision-making processes in pediatric HSCT. A narrative approach was selected due to the heterogeneity of the available evidence, which includes qualitative studies, ethical analyses, and observational research with diverse methodologies, limiting the feasibility of a systematic review.

Electronic database searches were performed using PubMed, Scopus, Embase, and Web of Science, including all available literature up to March 2024. The search strategy included combinations of Medical Subject Headings (MeSH) and relevant keywords such as hematopoietic stem cell transplantation, decision making, pediatric patients, family perspectives, and shared decision making. Boolean operators (AND/OR) were used to improve the sensitivity and specificity of the search.

Articles published in English or Spanish were considered eligible if they explored factors influencing decision-making in HSCT from the perspectives of patients, families, or healthcare professionals. Studies addressing ethical considerations, psychosocial aspects, and clinical decision-making in pediatric transplantation were included. Gray literature was not systematically included in this review.

Reference lists of the selected articles were also manually reviewed to identify additional relevant publications that may not have been captured during the initial database search. The included studies were analyzed using a thematic analysis within a narrative synthesis approach to systematically identify and organize recurring themes related to clinical, social, and ethical factors influencing decision-making in pediatric transplantation. This review provides a descriptive synthesis of the literature and does not propose a formal conceptual framework or validated model, given the heterogeneity of the available evidence. No statistical synthesis (e.g., meta-analysis or meta-regression) was performed, as the heterogeneity of the included studies precluded quantitative analysis.

Factors influencing decision-making

Diagnosis

Parents of children with non-malignant diseases consider multiple factors when deciding whether to proceed with HSCT, including the child’s quality of life, recent complications of the disease, the need to make significant medical decisions, and concerns about future severe complications. Additional considerations include potential social isolation, changes in family dynamics, the severity of the disease, and the availability of compatible donors [6-8].

In contrast, patients and families dealing with malignant diseases often perceive the decision to undergo transplantation as urgent and frequently consider it the only viable therapeutic option. In these situations, transplantation is commonly viewed as part of the initial treatment plan and represents a source of hope for survival despite the associated risks [9,10].

Other factors frequently mentioned by parents include the child’s preferences, emotional aspects such as fear of the child’s death, the practical implications of the procedure, the potential risk of infertility, and religious beliefs. When patients themselves are interviewed, the most commonly reported motivations include the possibility of reducing the frequency of hospitalizations, the perception that the treatment is relatively safe, and the belief that it may produce fewer adverse effects compared with alternative therapies [11-13].

Information Availability and Understanding

Access to accurate and comprehensible information is a critical component of the decision-making process for patients and families. Patients with malignant diseases often face significant challenges in understanding the medical information provided, particularly because discussions are frequently framed around life-threatening conditions and urgent treatment decisions. In some cases, particularly in urgent clinical scenarios, the processing of information may extend into the peri-transplant or post-transplant period, as has been described in studies highlighting the challenges families face in understanding complex and high-stakes medical information [14].

In contrast, patients with non-malignant diseases and their families typically require more extensive information before making a decision. Many families actively search for additional information through external sources such as the internet and subsequently consult healthcare professionals to clarify the information they have found [9,10]. In some cases, information obtained online may influence treatment decisions or even lead families to question medical recommendations.

For this reason, effective communication between healthcare professionals and families is essential. Transparency, accessibility of information, and willingness to address questions are key elements that help maintain trust and strengthen the physician-patient relationship [15].

Socioeconomic Factors

Socioeconomic considerations also play a significant role in the decision-making process. The cost of transplantation, the patient’s educational situation, family finances, employment stability of parents or caregivers, and changes in family routines are among the most important factors influencing treatment decisions [9,16]. Families frequently express concerns about disruptions in work schedules, school activities, and daily life associated with the transplantation process.

Although religious beliefs, potential hair loss, and infertility may influence decisions, these factors have generally been reported as less influential than financial and logistical concerns [9,16]. Understanding the broader social context of families is therefore essential. The ethical framework of care emphasizes that the more healthcare professionals understand the lived experiences of patients and their families, the better they can determine what constitutes appropriate care in specific circumstances [17].

Religious and Spiritual Considerations

Religion and spirituality may play an important role in how adolescents and their families cope with illness and the transplantation process. Spiritual beliefs often influence how patients interpret their illness, perceive risks, and approach medical decision-making [18]. Addressing the spiritual needs of patients and families can therefore be an important component of comprehensive medical care.

Understanding the role of faith in patients’ lives allows healthcare professionals to engage in more informed discussions about treatment options and demonstrates respect for the religious values and cultural backgrounds of families undergoing transplantation [19].

Risk-Benefit Perception

Regardless of the specific indication for transplantation, most patients and families perceive HSCT as a potentially curative option that can significantly improve survival and quality of life. However, concerns about treatment failure, mortality, treatment-related complications, graft-versus-host disease, and risks to sibling donors are commonly expressed [9,16].

A realistic assessment of the probable outcomes of medical treatment based on the stage of the child’s disease is essential when considering transplantation, whether the therapeutic goal is curative or palliative [5]. Fertility preservation is another important issue, particularly for parents of prepubertal children. Healthcare professionals should inform patients and families about potential reproductive risks and available fertility preservation options before initiating gonadotoxic therapies [6,20]. Despite its importance, fertility counseling is not consistently addressed in clinical practice, highlighting the need for increased awareness and systematic integration into pre-transplant discussions. However, studies have shown differences in how fertility counseling is perceived by adolescents and their parents. For some adolescents, fertility considerations may represent a secondary concern compared with the immediate need to treat the underlying disease [6,21,22].

Stakeholders involved in decision-making

Patient Perspective

The voices of children and adolescents are increasingly recognized as important in treatment decision-making processes. When cognitively capable, pediatric patients should be given the opportunity to participate in discussions regarding their treatment options [9]. Studies have demonstrated that interventions promoting shared decision-making, such as decision aids, can significantly reduce decisional conflict among pediatric patients and their families when choosing treatment strategies [16,23].

Research also indicates that children and adolescents undergoing transplantation often want to receive information about their condition and participate in conversations about treatment decisions. When patients feel excluded from the decision-making process or inadequately informed, they are more likely to experience higher levels of decisional conflict [16,23].

Patients with malignant diseases or conditions such as Fanconi anemia often rely heavily on physicians for information and guidance regarding treatment options. In contrast, patients with non-malignant conditions may place greater trust in personal experiences or testimonies from other patients and families who have undergone transplantation [9].

Adolescence is characterized by ongoing neurodevelopmental changes, including an imbalance between the socioemotional system, which involves limbic and paralimbic structures, and the cognitive control system located in the prefrontal and parietal cortices. The socioemotional system tends to mature earlier, which may lead adolescents to prioritize immediate rewards over long-term consequences. These neurodevelopmental characteristics have important implications for medical decision-making in adolescents [24].

To determine whether an adolescent has the capacity to participate in healthcare decisions, several abilities must be assessed, including the ability to communicate a choice, understand relevant information, appreciate the consequences of the decision, and evaluate information rationally. In certain circumstances, adolescents may make their own healthcare decisions, such as when they are legally emancipated, when seeking treatment for sexually transmitted infections or substance abuse, or when considered “mature minors,” although legal definitions and requirements vary across jurisdictions [24].

Parental Perspective

In pediatric medicine, parents typically serve as the primary decision-makers for their children. Studies suggest that parents’ ability to cope with the potential loss of a child is one of the most influential factors affecting their treatment decisions. This emotional interconnection between parents and their children highlights the complexity of decision-making in life-threatening pediatric conditions [17].

Support from partners and extended family members has been identified as a crucial factor that helps parents cope with the emotional burden of making medical decisions for their children [16,25]. Healthcare professionals also play an important role in helping parents anticipate their children’s emotional responses and providing appropriate psychological resources, particularly for children who may experience high levels of anxiety or sadness during the treatment process [26].

When acting as surrogate decision-makers, parents may rely on several ethical frameworks, including pure autonomy, substituted judgment, and the best interest standard. Pure autonomy refers to situations in which the decision-maker knows the patient’s explicit wishes and preferences. Substituted judgment involves making decisions based on what the patient would have wanted, while the best interest standard focuses on determining which option provides the greatest overall benefit for the patient based on available medical information and prognosis [27].

Among the factors considered by parental decision-makers are the desire to maximize survival, trust and hope in a favorable outcome, the desire to avoid future regret, the goal of achieving the greatest therapeutic benefit, and the intention to respect the wishes of the child whenever possible. However, in end-of-life situations, parents may rely more heavily on physicians’ recommendations while hoping to achieve the best possible outcome for their child [28,29].

Decision aids have been developed to support shared decision-making by helping families understand treatment options, clarify risks and benefits, and align medical decisions with personal values and preferences [8].

Healthcare Professional Perspective

For patients undergoing HSCT, the distinction between providing benefit and avoiding harm can sometimes be unclear. Treatments that offer potential improvement often carry substantial risks, including the possibility of severe complications or death. Conflicts may arise between healthcare providers and families regarding the initiation or continuation of certain medical interventions.

Physicians are not ethically obligated to offer or perform every possible treatment option. Instead, medical interventions must be evaluated in collaboration with other healthcare professionals and within the broader context of the patient’s overall condition and the likelihood of achieving the intended goals of care. Both the Society of Critical Care Medicine and the American Academy of Pediatrics state that physicians are not required to offer therapies that are unlikely to achieve the patient’s care objectives [5,9].

Specific medical considerations influencing transplantation decisions in diseases such as sickle cell disease include the patient’s age, donor human leukocyte antigen compatibility, and the presence of severe complications such as stroke or the need for chronic transfusions [30].

Structured decision-making approaches have been shown to improve both the quality of care and communication within transplant teams by establishing clearer clinical criteria, defining post-transplant responsibilities, and incorporating psychological evaluation before transplantation [31].

Nursing staff also play a fundamental role within the multidisciplinary transplant team. Nurses sometimes perceive physicians as overly optimistic when discussing prognosis with patients and families, potentially offering more hope than the clinical situation may justify. While maintaining hope can help patients endure difficult treatments, excessive optimism may raise ethical concerns if it fails to fully reflect the medical realities of the situation [17].

Therefore, healthcare professionals have an important moral responsibility to advocate for the best interests of the child while carefully reflecting on the limits of medical treatment. Clear, compassionate, and respectful communication remains essential in guiding therapeutic decision-making in pediatric transplantation [31].

Discussion

Decision-making in pediatric HSCT is a complex process influenced by clinical uncertainty, the social context in which decisions are made, and the perspectives of multiple stakeholders involved in the care of the child. The complexity of underlying diseases, the urgency of treatment decisions, the economic implications of transplantation, and the difficulty in predicting individual outcomes make these decisions particularly challenging for both families and healthcare professionals [4].

Previous studies have emphasized the importance of structured decision-making processes that support families throughout the treatment journey. Models incorporating elements such as communication, empathy, compassion, and quality of care have been proposed to facilitate informed and ethically sound decisions. These approaches underscore the need for dynamic and interactive processes in which medical information, family values, and psychosocial factors are continuously integrated [13,32,33].

In pediatric settings, decision-making responsibility generally rests with parents or legal guardians, who are presumed to act in the best interests of the child. However, conflicts may arise when parental decisions could place the child at significant risk. In such situations, healthcare professionals may intervene after attempts at shared decision-making have failed, including involving child protection services or seeking judicial authorization when necessary [24,34,35].

Respect for the child’s developing autonomy is reflected in the concept of assent. Although children may lack full legal capacity to provide informed consent, involving them in age-appropriate discussions allows them to express preferences and participate in decision-making processes. This approach reinforces ethical practice while acknowledging developmental limitations [24,34,35].

End-of-life considerations further complicate decision-making in pediatric HSCT. The emotional weight of childhood mortality, combined with the unpredictable clinical course, makes it difficult to distinguish between potentially beneficial interventions and medically futile treatments. Patients may undergo multiple intensive care admissions, where decisions regarding continuation or limitation of therapy are particularly complex [32].

Concerns about treatment futility and quality of life are central to these decisions. However, objectively assessing quality of life remains challenging due to the multidimensional nature of outcomes in critically ill pediatric patients, further complicating clinical and ethical decision-making [36].

Limitations

This narrative review has some limitations. As a narrative synthesis, the selection of studies may be subject to selection bias and does not follow the structured methodology of a systematic review. To mitigate this risk, multiple databases were searched, predefined inclusion criteria were applied, and reference lists of selected articles were manually reviewed to identify additional relevant studies. In addition, heterogeneity in study design and patient populations among the included studies may limit the generalizability of some findings. This heterogeneity includes variability in underlying diseases (malignant vs. non-malignant conditions), transplant modalities (autologous vs. allogeneic HSCT), and differences in patient age groups and healthcare settings. Nevertheless, this review provides a comprehensive overview of the ethical, clinical, and social factors influencing decision-making in pediatric HSCT.

## Conclusions

Decision-making in pediatric HSCT is a complex and multifactorial process influenced by clinical prognosis, uncertainty regarding outcomes, socioeconomic context, and the perspectives of multiple stakeholders. Patients, parents, and healthcare professionals all contribute to shaping treatment decisions, highlighting the importance of shared decision-making models. Clear communication, realistic expectations, and psychological support are essential components that help families navigate these difficult choices. Understanding the ethical, emotional, and social dimensions involved in pediatric transplantation is fundamental to improving patient-centered care and supporting families facing high-stakes medical decisions.

## References

[1] Balassa K, Danby R, Rocha V (2019). Haematopoietic stem cell transplants: principles and indications. Br J Hosp Med (Lond).

[2] Barriga F, Ramírez P, Wietstruck A, Rojas N (2012). Hematopoietic stem cell transplantation: clinical use and perspectives. Biol Res.

[3] Sastre-Urgellés A (2006). Trasplante de progenitores hematopoyéticos. An Pediatr Contin.

[4] Bendorf A, Kerridge IH (2011). Ethical issues in bone marrow transplantation in children. J Paediatr Child Health.

[5] Needle J (2014). Ethical challenges with the advancement of hematopoietic stem cell transplant. J Pediatr Intensive Care.

[6] Sinha CB, Meacham LR, Bakshi N, Ross D, Krishnamurti L (2023). Parental perspective on the risk of infertility and fertility preservation options for children and adolescents with sickle cell disease considering hematopoietic stem cell transplantation. Pediatr Blood Cancer.

[7] Sinha CB, Bakshi N, Ross D, Loewenstein G, Krishnamurti L (2021). Primary caregiver decision-making in hematopoietic cell transplantation and gene therapy for sickle cell disease. Pediatr Blood Cancer.

[8] Sullivan KM, Horwitz M, Osunkwo I, Shah N, Strouse JJ (2018). Shared decision-making in hematopoietic stem cell transplantation for sickle cell disease. Biol Blood Marrow Transplant.

[9] Schulz GL, Kelly KP, Holtmann M, Doering MM, Armer JM (2018). Decision making in pediatric hematopoietic cell transplantation: influential factors vary among diseases. Pediatr Blood Cancer.

[10] Schulz G, Patterson Kelly K, Shenoy S, Armer J (2015). The perspectives of stakeholders in pursuing hematopoietic stem cell transplantation in children and adolescents: an integrative review. Biol Blood Marrow Transplant.

[11] Hankins J, Hinds P, Day S, Carroll Y, Li CS, Garvie P, Wang W (2007). Therapy preference and decision-making among patients with severe sickle cell anemia and their families. Pediatr Blood Cancer.

[12] Pelletier W, Hinds PS, Alderfer MA, Fairclough DL, Stegenga K, Pentz RD (2014). Themes reported by families as important when proceeding with pediatric hematopoietic stem cell transplantation. Pediatr Blood Cancer.

[13] Nickoloff S, Marks S (2012). Opioid induced neurotoxicity in a patient with cancer related pain. J Pain Symptom Manage.

[14] Khemani K, Ross D, Sinha C, Haight A, Bakshi N, Krishnamurti L (2018). Experiences and decision making in hematopoietic stem cell transplant in sickle cell disease: patients' and caregivers' perspectives. Biol Blood Marrow Transplant.

[15] Sharpe K, Di Pietro N, Jacob KJ, Illes J (2016). A dichotomy of information-seeking and information-trusting: stem cell interventions and children with neurodevelopmental disorders. Stem Cell Rev Rep.

[16] Schulz GL, Kelly KP, Holtmann M, Armer JM (2021). Navigating decisional conflict as a family when facing the decision of stem cell transplant for a child or adolescent with sickle cell disease. Patient Educ Couns.

[17] Dierckx de Casterlé B, Verhaeghe ST, Kars MC (2011). Researching lived experience in health care: significance for care ethics. Nurs Ethics.

[18] Ragsdale JR, Hegner MA, Mueller M, Davies S (2014). Identifying religious and/or spiritual perspectives of adolescents and young adults receiving blood and marrow transplants: a prospective qualitative study. Biol Blood Marrow Transplant.

[19] Ragsdale JR, Othman M, Khoury R (2018). Islam, the Holy Qur'an, and medical decision-making: the experience of Middle Eastern Muslim families with children undergoing bone marrow transplantation in the United States. J Pastoral Care Counsel.

[20] Loren AW, Senapati S (2019). Fertility preservation in patients with hematologic malignancies and recipients of hematopoietic cell transplants. Blood.

[21] Barnbrock A, Hamannt F, Salzmann-Manrique E, Rohm T, Lange S, Bader P, Jarisch A (2023). Look at the future -perceptions of fertility counseling and decision-making among adolescents and their parents in the context of hematopoietic stem cell transplantation-experience of one major center for pediatric stem cell transplantation. Front Pediatr.

[22] Ginsberg JP, Li Y, Carlson CA (2014). Testicular tissue cryopreservation in prepubertal male children: an analysis of parental decision-making. Pediatr Blood Cancer.

[23] Mekelenkamp H, Smiers F, Camp N, Stubenrouch F, Lankester A, de Vries M (2021). Decision making for hematopoietic stem cell transplantation in pediatric, adolescent, and young adult patients with a hemoglobinopathy-shared or not?. Pediatr Blood Cancer.

[24] Diekema DS (2020). Adolescent brain development and medical decision-making. Pediatrics.

[25] Son H, Oyesanya TO, Brandon D, Docherty SL (2023). Challenges and coping between parents in shared decision-making for children with complex, life-threatening conditions: a qualitative content analysis. J Pediatr Nurs.

[26] Moore CW, Rauch PK (2006). Addressing parenting concerns of bone marrow transplant patients: opening (and closing) Pandora's box. Bone Marrow Transplant.

[27] Rogers CG, Duhon G (2009). Surrogate decision making for the previously capable minor. Clin Pediatr (Phila).

[28] Mekelenkamp H, Lankester AC, Bierings MB, Smiers FJ, de Vries MC, Kars MC (2020). Parental experiences in end-of-life decision-making in allogeneic pediatric stem cell transplantation: "have I been a good parent?". Pediatr Blood Cancer.

[29] Hamilton JG, Hutson SP, Moser RP, Kobrin SC, Frohnmayer AE, Alter BP, Han PK (2013). Sources of uncertainty and their association with medical decision making: exploring mechanisms in Fanconi anemia. Ann Behav Med.

[30] Krishnamurti L (2021). Hematopoietic cell transplantation for sickle cell disease: updates and future directions. Hematology Am Soc Hematol Educ Program.

[31] Silva AF, Kosmaliski UL, Antunes BS, Motta MD (2022). Allogeneic hematopoietic stem cell transplantation in children and adolescents: ethical problems faced by the multidisciplinary team. Rev Gaucha Enferm.

[32] Hill M, Lewis C, Riddington M (2019). Stakeholder views and attitudes towards prenatal and postnatal transplantation of fetal mesenchymal stem cells to treat osteogenesis imperfecta. Eur J Hum Genet.

[33] Baker JN, Barfield R, Hinds PS, Kane JR (2007). A process to facilitate decision making in pediatric stem cell transplantation: the individualized care planning and coordination model. Biol Blood Marrow Transplant.

[34] Spriggs M (2023). Children and bioethics: clarifying consent and assent in medical and research settings. Br Med Bull.

[35] Nickels AS, Myers GD, Johnson LM, Joshi A, Sharp RR, Lantos JD (2016). Can parents refuse a potentially lifesaving transplant for severe combined immunodeficiency?. Pediatrics.

[36] Hopkins S (2005). Withholding or withdrawing intensive care treatment in paediatric bone marrow transplant: a moral maze. Eur J Oncol Nurs.

